# Ictal epileptic headache revealing non convulsive status epilepticus in a case of eyelid myoclonia with absences

**DOI:** 10.1186/s10194-015-0587-4

**Published:** 2015-12-07

**Authors:** Martina Fanella, Alessandra Morano, Jinane Fattouch, Mariarita Albini, Sara Casciato, Mario Manfredi, Anna Teresa Giallonardo, Carlo Di Bonaventura

**Affiliations:** Department of Neuroscience, Neurology Unit, “Sapienza” University, Rome, Italy

**Keywords:** Eyelid myoclonia with absences, Ictal epileptic headache, Cortical spreading depression, Comorbidity

## Abstract

Epileptic seizures and headache attacks are two common neurologic phenomena characterized by paroxysmal alteration of brain functions followed by complete restauration of the baseline condition. Headache and epilepsy are related in numerous ways, and they often co-occur. Although the link between these two diseases is not completely clear, several clinical, physiopathological and therapeutic features overlap. Headache is reported in association with epileptic seizures as a pre-ictal, ictal or post-ictal phenomenon. We present the case of a 40 year-old woman affected by eyelid myoclonia with absences (EMA) with a history of prolonged headache attacks. A video-EEG recording performed during one of these episodes showed subcontinuous epileptic activity consisting of generalized spike-and-wave discharges (GSWDs), clinically associated with tensive headache. Our work represents one of the few well EEG-documented cases of ictal epileptic headache in idiopathic generalized epilepsy (IGE).

## Background

Epilepsy and headache are two common neurologic disorders with recurrent, transient, paroxysmal episodes of altered brain function, that share clinical features, pathophysiological mechanisms and therapeutic approaches. Epilepsy and headache may either coexist independently in the same individual or be related in numerous ways, although the nature of this association is unclear. Headache is common in epileptic patients and it can be defined as inter-ictal (not time-locked to a seizure) or peri-ictal (pre-ictal, ictal, post-ictal) headache [1]. When headache itself represents the “sole” epileptic manifestation, it is called ictal epileptic headache (IEH). The diagnosis of this rare phenomenon, characterized by cephalic pain, with or without migrainous features, relies on the EEG evidence of an irrefutable ictal pattern and on the electro-clinical responsiveness to intravenous antiepileptic drugs [2, 3]. In literature, there are few well documented cases of IEH in patients with focal seizures, whereas IEH is only exceptionally reported in generalized epilepsies [4, 5]. In this paper, we describe the case of a 40 year-old woman affected by eyelid myoclonia with absences (EMA) with a sustained episode of ictal epileptic headache documented by video-EEG study.

## Case presentation

**Fig. 1:**
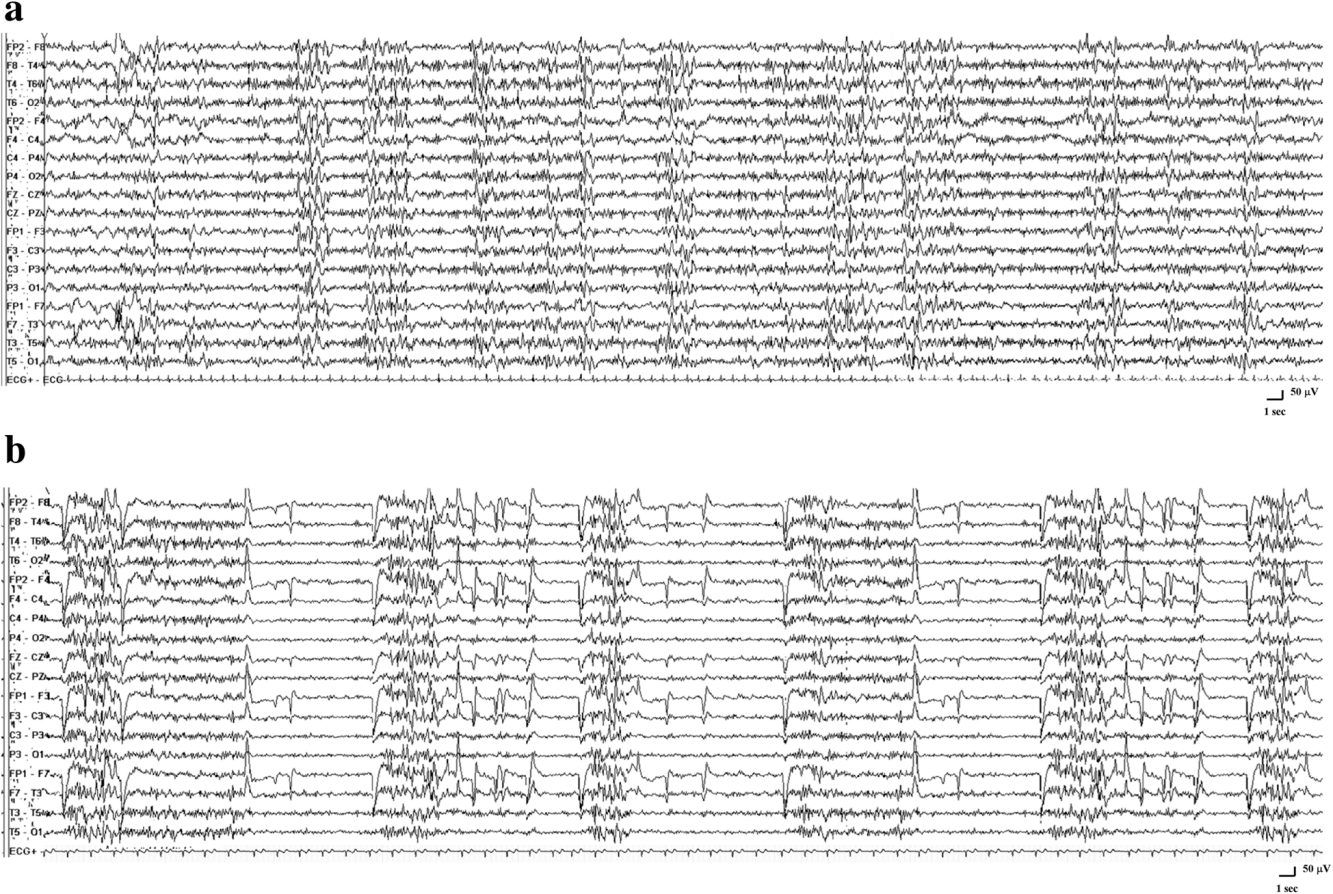
Ictal epileptic headache during NCSE documented by video-EEG. The EEG tracing showed the activation of subcontinuous epileptic activity consisting of GSWDs, clinically related to a prolonged tensive headache with bilateral and symmetrical eyelid flutter (**a**). The EEG pattern confirmed the presence of an eye closure sensitivity characterized by GSWDs related to bilateral and symmetrical eyelid flutter (**b**)

**Fig. 2:**
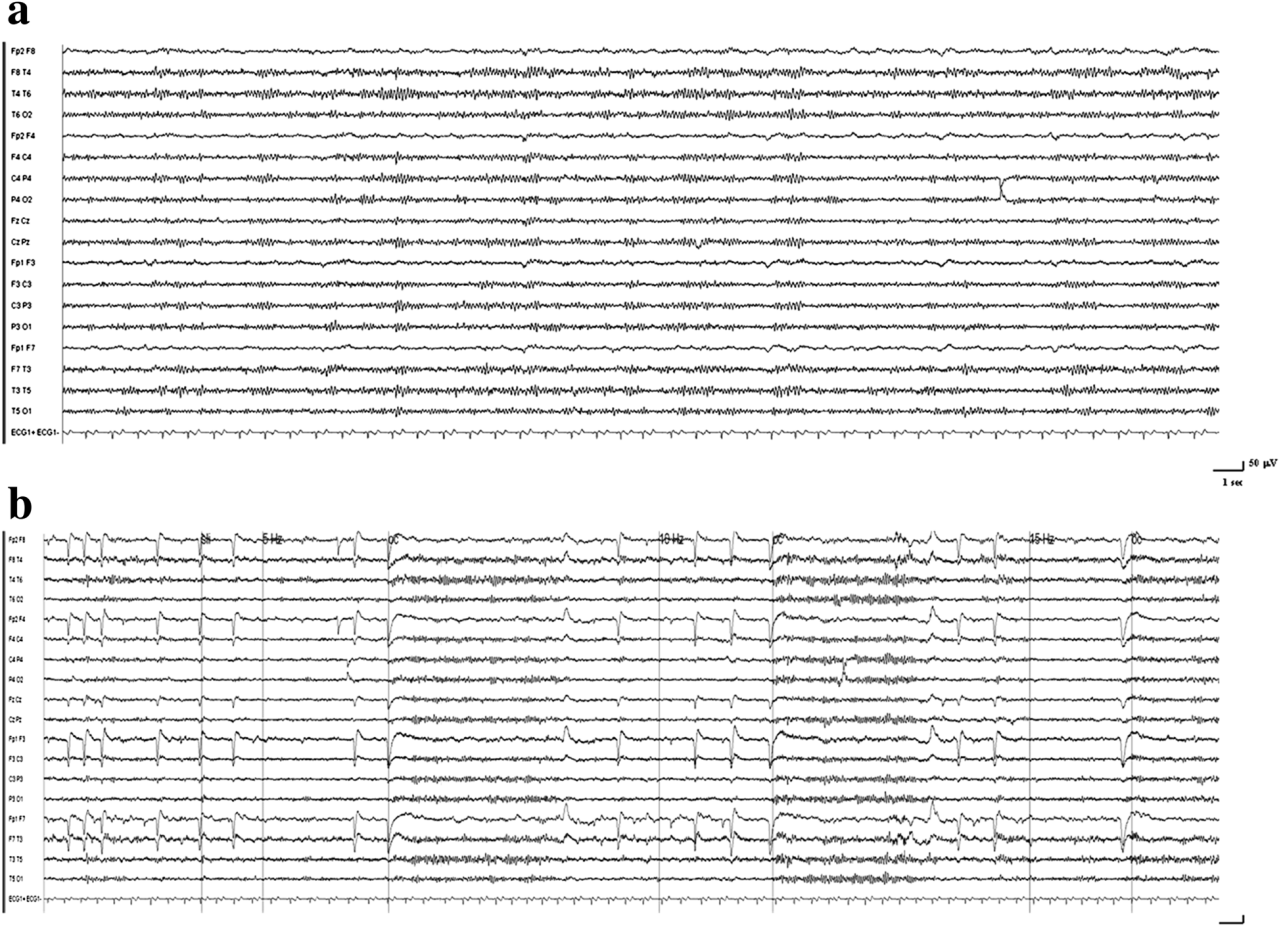
Video-EEG performed some days after NCSE, showing a normal tracing during rest (**a**) and during eye closure (**b**)

We report the case of a 40-year-old woman referred to our Epilepsy Unit for a history of seizures started at the age of 14, consisting of typical absences with or without eyelid flutter and tonic-clonic seizures. Her family history was positive for migraine without aura. Neurologic examination and neuroimaging study were normal. Several interictal EEG recordings, performed over time, documented the presence of generalized polyspike-and-wave complexes, triggered by active eye closure, often associated with eyelid myoclonia and photosensitivity. A diagnosis of eyelid myoclonia with absences (EMA) was made on the basis of electroclinical and neuroimaging findings. The patient was started on antiepileptic drugs, which led to seizure-freedom for about ten years. In addition, she had always presented sporadic, prolonged tensive headache attacks, rarely accompanied by migrainous features (nausea and visual manifestations). Recently, on waking, the patient complained the sudden onset of a sustained tensive headache, unresponsive to conventional treatment. Because of the persistence of symptoms, after about three hours she came to our attention, and we performed a video-EEG recording, which showed the presence of a subcontinuous epileptic activity consisting of generalized spike-and-wave discharges (GSWDs), clinically correlated with a tensive headache with bilateral and symmetrical eyelid flutter, facilitated by eye closure (Fig. 1). The electroclinical pattern lasted for hours, configuring a non-convulsive status epilepticus (NCSE). During video-EEG monitoring, Diazepam was administered with a gradual improvement of both clinical symptoms and EEG abnormalities. The patient reported complete resolution of the headache attack after about 24 h. Video-EEG recordings performed on the following days were normal (Fig. 2).

## Discussion and conclusions

The complex relationship between epilepsy and headache/migraine remains poorly understood. Although these two conditions often co-occur and are related in numerous ways, epidemiologic, genetic and pathophysiological studies are controversial. Besides, the prevalence and the incidence of these disorders are different in all stages of life. In epileptic patients the onset of the disease generally occurs under the age of one and over the age of sixty, while migraine/headache mainly involves people in their middle age. In young patients autonomic features during both headache/migraine attacks and seizures are more frequent when compared to the adult population, where autonomic manifestations are usually associated with other neurologic symptoms (the autonomic system threshold appears to be age-specific and, particularly in children, its activation is presumed to be lower than that of sensori-motor or other networks) [6, 7].

Assumed that interictal headache is the most frequent form (25–60 % of the cases), in the subgroup of patients with peri-ictal headache, post-ictal headache account for 10–50 %, pre-ictal for 5–15 % and ictal for 3–5 % [8]. According to IEH criteria [2], only 15 cases of IEH have been reported in literature so far; however, they were all characterized by extremely heterogeneous headache clinical features (in terms of pain characteristics and location), etiology and EEG pattern. Migraine with or without aura, tension-type or unclassifiable headache have been described. According to published data, IEH is mainly associated with partial epileptic syndromes or encephalopathies; from a subsyndromic point of view, most patients had a symptomatic posterior focal epilepsy. Only one case of IEH in idiopathic generalized epilepsy (IGE) has been reported until now [4, 5].

Our case offers the opportunity to document the rather exceptional association of IEH and EMA. The electroclinical pattern detected during video-EEG was characterized by tensive headache with bilateral and symmetrical eyelid flutter facilitated by eye closure, correlated with GSWDs. This epileptic activity persisted for hours representing, by definition, a NCSE.

The headache attack as expression of an epileptic seizure is an interesting phenomenon and its pathophysiological mechanisms have received considerable attention over the last decade.

Neocortical dysexcitability (hypo- and hyper-excitation in headache patients and hyper-excitation in epileptic patients) is the main physiopathological mechanism underlying the onset of both diseases. Cortical spreading depression (CSD) seems to be the connection point between headache and epilepsy. CSD represents a neuronal depolarization wave, followed by a suppression of bioelectrical activity. Neural modifications are associated with changes in regional blood flow, which increases during the phase of depolarization and lowers during neural suppression. The occipital cortex is considered to be the area where most migraine-related CSD phenomena start [9]. Moreover, the occipital visual cortex plays an important role in the genesis of eye closure–induced seizures and photosensitivity in several common forms of epilepsy, including EMA. Indeed, the interictal epileptiform discharges over the posterior brain regions and spiky posterior alpha activities suggest the main role of occipital cortex, in EMA network. The normal alpha generator produces the posterior alpha rhythm reactive to eye opening and closure (blinking). The malfunction of such generator may produce the spiky posterior alpha activity during sustained eye closure [10]. These electro-clinical data were corroborated by EEG-correlated functional magnetic resonance imaging (fMRI) studies in patients with EMA: an increased blood oxygen level-dependent (BOLD) signal in the visual cortex was detected after eye closure, regardless of the presence of EEG paroxysms, suggesting a transient state of increased neuronal activity or a defective inhibition [11]. Moreover, in photosensitive patients local hyperexcitability of the primary visual cortex has been detected, as well as impaired intracortical inhibition [12]. In some patients with IEH, intermittent photic stimulation (IPS) determined a photoparoxysmal response (PPR) with or without headache [13, 14]. In other words, the electro-clinical characteristics and focal changes of cerebral blood flow observed in patients with EMA suggest the main involvement of the occipital cortex. These findings, in agreement with the role of the occipital cortex during migraine-related CSD, may explain the occurrence of headache as the main ictal symptom of NCSE in our case. Although the putative role of the excitation/inhibition unbalance in the occipital cortex constitutes an intriguing issue, in IGE patients with IEH the speculation is strongly limited by the poor availability of well-documented cases.

To date, indeed, IEH remains an extremely rare phenomenon and thus the exact pathophysiological point of connection between headache and epilepsy remains enigmatic.

## Consent

Written informed consent was obtained from the patient for publication of this Case Report and any accompanying images. A copy of the written consent is available for review by Editor- in- Chief of this Journal.

## Abbreviations

IEH: Ictal epileptic headache
EMA: Eyelid myoclonia with absences
GSWDs: Generalized spike-and-wave discharges
NCSE: Non-convulsive status epilepticus
IGE: Idiopathic generalized epilepsy
CSD: Cortical spreading depression
fMRI: Functional magnetic resonance imaging
BOLD: Blood oxygen level-dependent
IPS: Intermittent photic stimulation
PPR: Photoparoxysmal response
